# Insights into Global Health Practice from the Agile Software Development Movement

**DOI:** 10.3402/gha.v9.29836

**Published:** 2016-04-29

**Authors:** David Flood, Anita Chary, Kirsten Austad, Anne Kraemer Diaz, Pablo García, Boris Martinez, Waleska López Canú, Peter Rohloff

**Affiliations:** Wuqu’ Kawoq ∣ Maya Health Alliance, Santiago Sacatepéquez, Guatemala

**Keywords:** implementation science, program design, global health, interventions, local

## Abstract

Global health practitioners may feel frustration that current models of global health research, delivery, and implementation are overly focused on specific interventions, slow to provide health services in the field, and relatively ill-equipped to adapt to local contexts. Adapting design principles from the agile software development movement, we propose an analogous approach to designing global health programs that emphasizes tight integration between research and implementation, early involvement of ground-level health workers and program beneficiaries, and rapid cycles of iterative program improvement. Using examples from our own fieldwork, we illustrate the potential of ‘agile global health’ and reflect on the limitations, trade-offs, and implications of this approach.

## Paper context

The gap between research-based evidence and the realities of implementation under real-world conditions is a major debate in global health. Here, we review the principles of agile design from software development, and we describe an adaptation of this framework to global health implementation science. This framework, which we call *agile global health*, will be of utility to other global health implementers seeking to design, deploy, and evaluate contextually rigorous global health programs.

## Introduction

In the field of global health delivery, a vigorous debate has recently emerged about the relationship between research and implementation. The rapid growth of the field of implementation science underscores how many health interventions supported by high-level research are either impractical to deliver at scale or have reduced effectiveness in the field (1, 2). Although the primary objective of implementation science is to bridge the gap between evidence and practice (3, 4), those who design global programs may still wonder how best to apply available evidence. This issue is especially relevant when working with populations that are very different from those in which interventions have been tested, when local health systems are idiosyncratic and unpredictable, or when planned interventions rely on behavior change in cross-cultural settings. Global health practitioners who build or support health delivery programs, a group we anticipate describes many readers of this journal, may feel frustration that the current models of global health research and implementation are overly focused on narrow interventions, slow to provide health services in the field, and relatively ill-equipped to respond to local contexts.

In this Current Debate, our objective is to consider the application of principles from a popular software design methodology known as *agile development* to the practice of global health. We first describe the foundations and core principles of agile software development, which we feel offers a flexible framework that is broadly applicable to any design process, including global health program design. The motivation for writing this viewpoint comes from our experiences as staff with a non-governmental organization in Guatemala that collaborates with rural, Mayan-speaking indigenous populations to build and sustain high quality health programs. Through examples from our own fieldwork, we illustrate the potential of ‘agile global health’ to overcome some of the challenges described above. We conclude by reflecting on the limitations, trade-offs, and implications of this approach.

## History and description of agile software development

Agile software development is a design methodology that emphasizes flexibility and adaptability; close collaboration between developers and end users; and short, iterative product cycles. Agile design unites several related software production strategies that emerged in the 1990s and became popular in the early 2000s (5). Recently, agile software development has become ‘successful beyond anyone's dreams … the buzz of the industry’ and is ‘not just a collection of software techniques but a movement, an ideology, a cause’(6).

Agile software development emerged from software engineers’ frustrations with traditional design methods that were perceived to be bureaucratic, inflexible, and cumbersome. These traditional methods were largely variations on the ‘waterfall’ model, where a project progresses downhill through structured phases of product conception, analysis, design, development, testing, and implementation. The waterfall model utilizes extensive up-front planning, delivers a functional product late in the project lifecycle, and incorporates feedback from end users in the final phase of the project. In critics’ eyes, the model assumes that problems are predictable and solvable through planning, and it is therefore not equipped to address unpredictability or adapt to changes in design needs.

In contrast, agile design models limit the amount of upfront planning. The goal is to produce usable software early in the process – even if it is only ‘minimally functional’ – and then make incremental improvements using many iterative cycles of testing, feedback, and redesign. In this way, input from end users is prioritized, and emphasis is placed on physical proximity and face-to-face communication between end users and designers from the very beginning. In addition, rather than attempting to resolve all problems and contingencies that could arise in a complex system through advanced planning, agile proponents prefer to expect and embrace changing design requirements. To foster adaptability and flexibility, the management style in agile development tends to be decentralized. Development teams include members with varying areas of expertise who are given independence to organize their work. Ultimately, advocates of agile design argue that constant user feedback, rapid product iterations, and an emphasis on design flexibility result in software that better meets the needs of its users.

## Agile software design in global health

We were first exposed to the principles of agile software design while implementing mobile technology to improve electronic medical documentation in rural Guatemala. We noticed that the interactions between software developers and end users (in our case, rural pregnant women and patients with chronic health care needs) helped us very quickly identify software problems, correct them, and then redeploy the improved technology. We were also impressed with patients’ and frontline health care workers’ enthusiasm for interacting with and providing feedback on early-stage technology.

The few published examples of agile design in global health contexts have, like our own experiences, been promising. For example, one case study used agile design concepts to build a mobile health tool to assess and manage cardiovascular disease in rural India (7). Before starting, the development team realized that a conventional needs assessment would be inadequate to understand how their users – rural health workers in India – would utilize the mobile tools in practice. Thus, the multidisciplinary team developed software prototypes that were tested in the field, immediately redesigned based on health worker feedback, and re-released. During this iterative process, unanticipated issues were solved in creative ways. For example, many patients did not know their age or date of birth, so the designers incorporated into the mobile tool a list of important historical events such as Indian Independence Day that could be used to estimate age.

## Towards agile global health

Reflecting on our experiences and those of others in implementing technology-based global health interventions using agile design has led us to realize that many of the ideas that govern agile software design are not necessarily specific to software development. Rather, they reflect sound principles of rapid, cooperative, and flexible design that are broadly applicable to any unpredictable work environment. As the settings in which we work in rural Guatemala are similarly idiosyncratic and dynamic, we began to apply the insights of agile design more broadly to our efforts to design and implement health programs. These experiences lead us to propose an agile design framework – *agile global health* – for use in global health practice.

First, we note that the implementation of most global health programs follows a process analogous to the waterfall model of traditional software development. Emphasis is placed on selecting interventions that are supported by high-level evidence and applying or scaling them within a specific setting. This approach requires a high degree of planning, analysis, and testing before any services are delivered. In the initial planning phase, the point at which the most crucial program decisions are made, the ‘users’ of the intervention generally have limited input. In later planning phases, pilot studies are utilized, but they are modest in scope relative to the size of the program as a whole, and they usually serve to confirm the program's feasibility and acceptability rather than to elicit feedback that could lead to major changes in design.

After implementation of the global health intervention has begun – a phase corresponding to the delivery of the final product in traditional software development – there is usually very limited flexibility to modify its design. The targeted nature of the program or intervention fosters the specialization of implementation teams, highly formalized work flows, and hierarchical organizational structures where both authority and information flow from the top (e.g. elite global or national policy makers) to the bottom (e.g. local health workers). Overall, as with early idealized process models in implementation science (8, 9), traditional global health program development tends to be stepwise and linear, and design and implementation are separated both temporally and spatially. Table 1 summarizes the differences between agile global health and these traditional approaches (10).

**Table 1:** Characteristics of agile global health

	Traditional global health program development	Agile global health program development
Fundamental assumption	Global health design problems can be predicted and resolved through upfront planning	Due to complexity and uncertainty within systems, design problems usually cannot be predicted and resolved through upfront planning
Management style	Vertical, centralized	Horizontal, decentralized
Teamwork philosophy	Teams are divided along areas of specific expertise	Teams are multidisciplinary and are given independence to organize their own work
Communication	Formal, with an emphasis on written documents and technical or research publications	Informal, with an emphasis on physical proximity and face-to-face communication
User's role	Important at the end of the process.	Critical throughout the process.
Project trajectory	Guided by sequential phases or funding cycles	Guided by iterative design cycles of testing, feedback, and redesign
Relationship between design and implementation	Separation of design and implementation phases	Integration of design and implementation

This table is inspired by a similar table in Ref. (10).

An agile global health approach, on the other hand, places the emphasis on *providing health services*. Elements of program design, workability, and acceptability are rapidly tested and iterated at precisely the same time and in the same space that tests of efficacy are being worked out. While committed to responsible prior planning, agile global health simultaneously recognizes the limits of advanced preparation in dynamic environments. It highly values the involvement of end users (patients or ground-level health workers) from the beginning of the design process, and it prioritizes the early delivery of health programs and services. An agile global health methodology uses iterative design cycles consisting of deploying preliminary programs in the field, getting feedback from users, redesigning the program, and redeploying the program in the field.

Intrinsic to this process is the fundamental assumption that global health settings are complex, unpredictable, and dynamic. As such, local particularities – the biological, social, linguistic, cultural, and environmental factors that global health science must respect – are integrated into the program development process *a priori* rather than viewed as barriers to implementation *a posteriori*. Just as agile software development was guided by a number of principles outlined in the Agile Manifesto, we propose principles of agile global health in Box 1. We see these principles as complementary to other rapid cycle methods from the fields of quality improvement and implementation science such as Plan-Do-Study-Act cycles (11) and the Knowledge to Action framework (12).

In our own organization, we are now actively working to restructure our program development initiatives along agile design principles. We have always employed ethnography, formative research, and participatory methods to guide the design and implementation of our programs within local contexts. However, we have concluded that although these community-engagement strategies generate important contextual knowledge, they are too divorced from the delivery of health services and, therefore, do not directly contribute to rapid cycle change. An ‘agile global health’ model can, however, provide this necessary framework. As an example, we describe how our diabetes programming has benefitted from this reframing of agile global health (Box 2).

*Box 1.* Principles of agile global health design

1. The ‘users’ of global health programs – that is, the patients and ground-level health workers – are involved in program development and implementation from the initial stages.
2. Valuable health services, even if limited in scope, should be delivered early in the design and implementation process.
3. The design and implementation of global health programs are tightly integrated; designers and users work together closely.
4. Field experience is critical for program designers in order to foster understanding of ground-level challenges.
5. Changes in the design of health programs are encouraged at any time, even major changes or changes late in the development process, if they make programs better.
6. The primary measure of progress is that health programs improve iteratively.
7. The highest priority is contextually rigorous health programs that serve the needs of specific communities and patients.

*Box 2.* Agile global health case study: designing diabetes programming in rural Guatemala

The burden of type 2 diabetes is rising rapidly in indigenous communities in Guatemala, yet quality diabetes care is largely unavailable (13). In 2010, we began offering diabetes clinical programming at the request of local indigenous leadership. We designed our initial program by adapting evidence-based chronic disease care models from other settings (14, 15) using extensive ethnographic research (16). As we began delivering diabetes services, however, we encountered many unanticipated challenges that necessitated program redesign:

- *Diabetes education*. We initially designed our diabetes education programming in a group-based format using an evidence-based curriculum rigorously adapted to the rural Mayan indigenous context (17). We chose a group-based design based on preliminary research suggesting that diabetes patients had high interest in group education (18), our previous success using this format within our maternal and child health programs, and a significant body of literature supporting the effectiveness of group- and peer-based models of diabetes self-management education (19, 20). When we implemented the program, however, we observed low attendance rates and a clear lack of interest in the sessions. Participants informed us that despite their initial excitement, they were unable to attend scheduled classes due to time and travel constraints. We quickly changed strategies and piloted home-based nurse visits, which led to a dramatic increase in participation.
- *Diabetes staffing model*. Based on an emerging literature from other settings showing the promise of community health workers (CHWs) within diabetes programs (21), we initially utilized CHWs to deliver the bulk of diabetes services. However, despite our efforts to aggressively train and support CHWs, we observed that in practice many diabetes patients were too medically complex to be safely managed by CHWs. Observing the success of local indigenous nurses in providing women's health services, we redesigned our model using nurses as primary diabetes providers. Internal data show that this model delivers excellent outcomes, is financially sustainable, and is easily scalable in our setting given the surplus of skilled indigenous nurses on the labor market.
- *Insulin prescriptions and adherence*. Our formative research indicated that patients with poorly controlled diabetes faced significant financial barriers to insulin (16), a finding in line with global literature primarily viewing access to insulin as problem of affordability and availability (22). Using published guidance (23), we thoughtfully developed a procurement and cold-chain infrastructure to purchase low-cost generic insulin from urban suppliers and distribute it to rural clinics. We also designed our clinical protocol to carefully dispense insulin and alleviate common psychological barriers to insulin (24). However, numerous patients refused insulin prescriptions once access barriers were overcome for complex, culturally informed psychosocial reasons. We subsequently redesigned our insulin strategy to incorporate patient testimonials about insulin, to emphasize family support during insulin initiation, and to make serial home visits to follow up on insulin use – strategies that dramatically increased the proportion of patients receiving insulin.

Our diabetes program provides several examples of challenges that could not have been anticipated despite rigorous selection of evidence-based interventions, formative research, and pilot testing. Rather, from an agile design perspective, designing our program at the same time as a ‘real-world’ deployment allowed us to uncover and respond to these challenges. Moreover, the close integration between program designers, front-line workers, and patients permitted major changes to occur at all stages of the program's development process. A keystone of our current diabetes programmatic structure is frequent, close contact between program managers and ground-level staff and patients, in line with agile design principles. This system has allowed the program to be iteratively improved and adapted to the rural Mayan setting in Guatemala.

## Conclusions

The agile global health approach resonates with calls from other commentators to ‘pu[t] practice into research (25)’, to engage communities in research (26–28), and to foster operations research within local systems (29). Taken together, we see these pleas as reflective of the broader tension in global health delivery between scale and context. Analogizing a framework for global health delivery from the agile software development movement may help resolve some of this tension, building on existing models and frameworks from the fields of quality improvement and implementation science. As we propose here, agile global health is a practical approach for developing health programs that work for specific communities or populations. Programs designed using this approach may not be themselves generalizable to other communities or populations. However, agile principles – tight integration between research and implementation, early involvement of users, and rapid cycles of iterative improvement – are broadly applicable in diverse settings.

At the same time, the agile design framework has its own limitations, which we continue to explore and encourage others to do as well. One such limitation is working out how agile design principle might operate within a rubric of health system strengthening involving many stakeholders. Another is that an agile design approach to global health delivery may tend to emphasize practical workability and speed of delivery over scientific precision, a trade-off that is likely not be appropriate for all global health programs or all implementers. Nevertheless, our experience providing clinical care and public health programming in very marginalized settings in Guatemala has convinced us of the potential benefits of the framework.
